# Pattern of vitreo-retinal diseases at University of Gondar tertiary eye care and training center, North-West Ethiopia

**DOI:** 10.1371/journal.pone.0267425

**Published:** 2022-04-21

**Authors:** Dagmawi Abebe, Asamere Tsegaw

**Affiliations:** Department of Ophthalmology, College of Medicine and Health Sciences, University of Gondar, Gondar, Ethiopia; Pusat Perubatan Universiti Kebangsaan Malaysia, MALAYSIA

## Abstract

**Objective:**

Vitreoretinal diseases are common causes of ocular morbidities and blindness. Data on the spectrum of vitreoretinal diseases needs to be studied and known in order to establish appropriate vitreoretinal care setups. The aim of this study was to determine the patterns of vitreoretinal diseases among patients who visited the vitreoretina clinic of University of Gondar Tertiary Eye Care and Training Center, NW Ethiopia (UoG-TECTC).

**Methodology:**

A hospital based cross sectional study was conducted from October/2017-September/2018. All patients who visited the vitreoretinal clinic for the first time during the study period were studied. Data were collected with standardized data extraction format entered into SPSS statistical package Version 20 and analyzed.

**Result:**

A total of 739 new patients who visited the vitreoretinal clinic were included in the study. The mean age was 50.26 +/- 19 years. The age group between 21–60 years accounted for 59.7% of study patients. Male’s accounted for 63.1% and 58.7% of the participants were from urban areas. Bilateral disease was diagnosed in 504 (68.2%) of patients and 220 (29.7%) were bilaterally blind at presentation. Three hundred eighty nine (52.6%) of them had duration of illness six months and above.

Diabetic Retinopathy (DR), Age Related Macular Degeneration (AMD) and Rhegmatoginous Retinal Detachment (RRD) were the top three retinal diseases accounting for 21.3%(196), 17.3% (128) and 12.4% (92) of diagnoses respectively. Systemic comorbidities were found in 44% (325) of the patients with diabetes mellitus, hypertension and hyperlipidemia being the commonest, occurring in 27.8%, 6.3% and 2.8% of study patients respectively. Cataract was the commonest ocular comorbidity seen in 33.5% of study participants.

**Conclusion:**

Vitreoretinal diseases affected a significant number of patients presented to our center and most of the study patients presented late with significant vision loss and blindness. Males were affected more than females and the age group between 21–60 years accounted nearly two-third of study patients. This is the working age group suffering from vision loss from vitreoretinal diseases. DR, AMD and RRD were the commonest retinal pathologies accounting for nearly half of the vitreoretinal diseases and these conditions are treatable either surgically or medically. However, available facilities for the management of these diseases are not adequate at the center. Strengthening the vitreoretinal services of UoG-TECTC with relevant equipment is recommended.

## Introduction

Vitreoretinal diseases are one of the common causes of ocular morbidities and blindness in the adult population. In children vitreoretinal conditions are already the most common causes of childhood blindness worldwide. Population based studies revealed an overall prevalence of vitreoretinal diseases 8.56% with a range between 10.4% up to 21.02% for the 40 year and above age group [1,2].

Vitreoretinal diseases encompass a wide variety of disorders among which the commonest ones are diabetic retinopathy (DR) and Age related macular degeneration (AMD). DR is the fifth leading cause of visual impairment and blindness globally. It is also the most common cause of new cases of blindness among working age adults in the developed world. In developing countries the burden of DR is increasing due to an overall increment in the prevalence of diabetes mellitus. AMD accounts for 8.7% of the total blindness globally and is the third common cause of visual impairment globally. It is the primary cause of visual impairment in industrialized countries [3].

The spectrum of vitreoretinal diseases varies from country to country and even it also varies from region to region in the same country. In Europe due to the increment in older age individuals AMD is the commonest retinal disorder in which 26.3% of Europeans over the age of 60 are affected by AMD. Next to AMD; Diabetic eye diseases follow, in which 25% of European diabetic patients are affected [4].

The 1981 Nepal Blindness Survey reported vitreoretinal diseases as the third leading cause of bilateral blindness, second only to cataract and its complications [5]. A more recent Nepalese population-based study reported vitreoretinal diseases to be the second common cause of bilateral blindness, second only to cataract, and the most common cause among pseudophakics [6].

A study done in Pakistan showed diabetic retinopathy was the leading vitreretinal cause for patient presentation followed by retinal detachment and high myopia [7].

There are only few studies done in African eye care set ups that addressed this issue. A report from south western Nigeria showed that macular diseases were the leading reason of patient presentation to the retina clinic followed by hypertensive retinopathy and retinochoroiditis. A similar study in south eastern Nigeria reported the hospital based prevalence rate of vitreoretinal diseases was 8.1%, with diabetic retinopathy being the leading cause of patient presentation followed by hypertensive retinopathy and Age Related Macular Degeneration [8,9].

In Ethiopia there is only one study done in minillik-II tertiary eye care center which reported diabetic retinopathy was the major reason for patient presentation to the retina clinic followed by retinal detachment [10]. However, this study was conducted in 2001, over 20 years ago, and the findings may not reflect the current situation. Therefore, there is a need for a more recent information.

The spectrum and pattern of vitreoretinal diseases needs to be studied and known in order to plan and establish vitreoretinal subspecialty care facilities. Therefore, this study is conducted to determine the clinical pattern of vitreoretinal diseases in patients who were examined at UOG tertiary eye care and training center.

## Methods

### Study design and period

A hospital based cross-sectional study was conducted to determine the pattern of vitreoretinal diseases at University of Gondar tertiary eye care and training center from October 2017-September 2018.

### Study area

The study was conducted at University of Gondar Tertiary Eye Care and Training Center, a major eye care and training center in Ethiopia. It is an ophthalmic referral center for an estimated 14 million people living in North-West Ethiopia. The center provides eye care services both at the base hospital and rural outreach sites and annually, over 80,000 patients are seen at both sites. The base hospital has 8 out-patient clinics, facilities for in-patient care with 30 beds and five operation theatres. Currently, the center has 10 ophthalmologists, five of them with subspecialty training. There are also 26 optometrists, 5 ophthalmic officers, and 29 general clinical nurses actively working in the outpatient clinics and operation theatres of the tertiary eye care and training center. The current student population of the center includes 26 Ophthalmology residents, over 200 BSC and MSC optometry students and 38 BSC ophthalmic Nursing students.

Available manpower, facilities and equipment for the management of vitreoretinal diseases at the center include, vitreoretina out-patient clinic, one vitreo-retinal consultant, Laser treatment room with Diod laser and Argon Green Laser equipments, Fundus Camera machine, four indirect ophthalmoscopes, B-scan ultrasound and facilities for intravitreal pharmacotherapy (Steroid and Anti-VEGF). There is no equipment for Scleral buckling and posterior vitrectomy surgeries.

### Study population

All patients with vitreoretinal diseases who visited the vitreoretina clinic of UOG tertiary eye care and training center for the first time during the study period were included in the study. Patient who had no vitreoretinal diseases, follow up patients and those with ocular media opacity for fundus evaluation were excluded from the study.

### Data collection procedure

Comprehensive Ophthalmic evaluation which included detailed demographic information, presenting complaint, associated ocular and systemic symptoms and past treatment history for ocular or systemic illnesses were taken for each patient. Best corrected visual acuity, intraocular pressure measurement and slit-lamp examination of the anterior and posterior segments were performed. Posterior segment evaluation was performed with +90D funduscopic lens on the slit-lamp for each patient by a vitreoretinal specialist after dilation of the pupil with 1% tropicamide eye drop. Indirect ophthalmoscopy with +20D lens was used to examine patients when indicated. Diagnosis was made based on clinical examination findings by a retina specialist. Pertinent and supportive laboratory investigations were also done for all patients. In some cases, there was more than one retinal diagnosis in one eye. For instance, an eye could have a retino-choroidal scar due to AMD as well as a branch retinal vein occlusion. On the other hand, both eyes could have different retinal diagnosis. For example, the right eye could have AMD and the left eye a central retinal vein occlusion. Alternatively both right and left eyes could have a single diagnosis for example diabetic retinopathy in both eyes. Each of the diagnosis was recorded separately on the data extraction format. Information on co-existing systemic diseases known previously were obtained from patients themselves, or diagnosed after systemic work-up.

Grading of best corrected visual acuity for visual impairment and blindness was done using the WHO/ICD 10 categorization of visual impairment as follows:

Normal-Best corrected visual acuity better or equal to 6/18 in the better eye.

Moderate visual impairment-Best corrected visual acuity between 6/24 and 6/60 in the better eye.

Severe visual impairment-Best corrected visual acuity less than to 6/60 and better than or equal to counting fingers at 3m in the better eye.

Blindness-Best corrected visual acuity less than counting fingers at 3m distance in the better eye.

#### Data analysis procedure

The collected data was checked for accuracy, coded and entered in to SPSS Version 20 (IBM Corp. Armonk, NY, USA). and descriptive analysis done. Age categories and other demographic variables, symptom types and duration, retinal diagnoses, and associated systemic disease were summarized as frequency and percentage. Category of presenting visual acuity was summarized as frequency and percentages.

The study was conducted in full compliance with the 1964 Helsinki Declaration on research involving human subjects. Prior to commencement of the study, ethical clearance was obtained from the Ethics Committee (Institutional Review Board) of the University of Gondar. All study participants gave written informed consent and were given a choice not to participate if they chose not to. Consent from minors was obtained from a parent or guardian who was required to give a written informed consent. All ages were included (there was no exclusion based on age).

## Results

A total of 739 patients who visited the vitreo-retina clinic of University of Gondar tertiary eye care and training center for the first time during the study period were studied. The mean age of the participants was 50.26 +/-19.36 years and median 53 (ranges 1 to 91) years. Males were predominant accounting 466 (63.1%) with a male to female ratio 1.7: 1 and 434(58.7%) of the participants were from urban areas. More than half of study patients, 441 (59.7%), were aged between 21 and 60 years and 270 (36.5%) were farmers [Table 1].

**Table 1:** Socio-demographic characteristics of patients with vitreo-retinal diseases presented to the University of Gondar Tertiary eye care and training center vitreoretina clinic, NW Ethiopia (n = 739).

variables	Categories	Frequency (%)
Gender	Male	466(63.1%)
	Female	273 (36.9%)
Age (years)	≤20	66(9%)
	21–60	441(59.7%)
	>60	337 (32.1%)
Occupation	Farmer	269(37%)
	Gov’t employee	174(23.4%)
	student	64(8.7%)
	Private employee	55(7.4%)
	Merchant	40(5.4%)
	Other	137(19%)
Marital status	Married	526(71.2%)
	Single	130(17.6%)
	Divorced	83(11.2%)
Religion	Christian	627 (84.8%)
	Muslim	112(15.2%)
Annual Income (USD)	<200	455(61.6%)
	200–600	167(22.6%)
	>600	117(15.8%)
Residence	Rural	305(41.3%)
	Urban	434(58.7%)

Most of the patients presented for the complaint of reduction of vision accounting 519(70.2%) of the participants and 389(52.6%) of them had duration of illness six months and above. Two hundred twenty (29.7%), 112 (16.2%) and 181(24.5%) patients had bilateral blindness, severe visual impairment and moderate visual impairment at presentation respectively.

Systemic comorbidities were found in 325 (44%) of the patients and from these, the top three were diabetes mellitus, hypertension and hyperlipidemia detected in 206(27.8%), 47(6.3%) and 21 (2.8%) of participants respectively. Non-vitreoretinal ocular comorbidities were found in 633(85.7%) of the patients and cataract 248(33.5%), blepharitis 177(24%) and Glaucoma 52(7%) were the top three comorbidities diagnosed in the study participant [Table 2]. Among the 739 patients, 504(68.2%) of them had bilateral disease and 158 (21.2%) patients had 2 or more different vitreoretinal diagnoses, the rest 581 (78.8%) had single vitreoretinal diagnosis giving a total of 918 vitreoretinal diagnoses.

**Table 2:** Clinical characteristics of patients with vitreo-retinal diseases presented to the University of Gondar Tertiary eye care and training center vitreoretina clinic, NW Ethiopia. (n = 739).

Variables	Categories	Frequency (%)
Presenting complaint	Reduction of vision	519(70.2%)
	Flashlights and floaters	47 (6.35%)
	Came for routine check up	118(16%)
	Others	55(7.4%)
Duration of complaint	>1 year	*290(39*.*2%)*
	6 month -1 year	*99 (13*.*4%)*
	1month to <6month	*124(16*.*8%)*
	< 1 month	*108(14*.*6%)*
Visual Acuity at presentation in the better eye	≥6/18	220(29.8%)
	6/24-6/60 (MVI)*	181(24.5%)
	<6/60-3/60 (SVI)*	112(16.2%)
	<3/60 (Blind)	220(29.7%)
Laterality	Unilateral disease	235(21.8%)
	Bilateral disease	504(68.2%)
Systemic co-morbodities	Diabetes Mellitus	206(27.8%)
	Hypertension	47(6.3%)
	Hyperlipidemia	21 (2.8%)
	Neurologic diseases	19(2.6%)
	Cardiac diseases	14(1.9%)
	Pulmonary diseases	13 (1.8%)
	HIV/AIDS	12(1.6%)
	Others	23(3.1%),
Ocular comorbidities	Cataract	246(33.5%)
	Blepharitis	173 (24%)
	Glaucoma	52 (7%)
	Refractive error	35(4.7%)
	others	97 (13%)

*MVI-Moderate Visual impairment.

*SVI-Severe visual impairment.

Among the group of vitreoretinal diseases the top three were retinal vascular diseases, macular diseases and retinal detachments accounting 304 (33.1%), 224 (24.4%) and 92 (10%) of the diagnoses respectively. Diabetic retinopathy, hypertensive retinopathy and retinal vein occlusions were the top three retinal vascular diseases diagnosed in 196(21.3%), 49(5.3%) and 45(5%) of diagnoses respectively. Age related macular degeneration was the commonest macular disease accounting 128 (17.3%) of diagnoses [Table 3]. The overall top three vitreo-retinal diseases were Diabetic Retinopathy, Age related macular degeneration and Rhegmatoginous retinal detachment accounting 196(21.3%), 128 (17.3%) and 77(8.3%) of diagnoses [Table 4].

**Table 3:** Category and vitreoretinal diagnoses made among patients presented to the University of Gondar Tertiary eye care and training center, vitreo-retina clinic, Ethiopia. (N = 918).

**Disease category**	**Diagnosis**	**Frequency (%)**
**Retinal vascular diseases**	Diabetic retinopathy	196(21.3%)
	Hypertensive retinopathy	49(5.3%)
	Branch or central Retinal vein occlusion	45(5%)
	Coats disease	3(0.3%)
	Idiopathic parafoveal telangiectesia	1(0.1%)
	**Sub-total**	**304 (33.1%)**
**Macular diseases**	Age related macular degeneration	128(14%)
	Macular edema of any cause	42(4.5%)
	Macular Hole	31(3.3%)
	Non-specific macular scar	14 (1.5%)
	Choroidal neovascularization (Non-AMD)	3 (0.3%)
	Macular dystrophies	3(0.3%)
	Epiretinal membrane	3(0.3%)
	**Sub-total**	**224 (24.4%)**
**Retinal detachments**	Rhegmatoginous retinal detachment	77(8.3%)
	Exudative Retinal detachment	4(0.4%)
	Tractional Retinal detachment	11(0.1%)
	**Sub-total**	**92 (10%)**
**Vitreous pathologies**	Vitreous hemorrhages of any cause	49 (5.3%)
	Posterior vitreous detachment	18(1.9%)
	Asteroid hyalosis	16(1.7%)
	others	4(0.4%)
	**subtotal**	**87 (9.4%)**
**Inflammatory/Infectious/Traumatic Chorioretinopathies**	Intermidiate uveitis	26(2.8%)
	idiopathic inflammatory retinochoroiditis	21(2.3%)
	Traumatic chorioretinopathies	20 (2.1%)
	Infectious retinochoroiditis	14(1.5%)
	Idiopathic Retinal vasculitis	8(0.9%)
	**Sub-total**	**89 (9.6%)**
Hereditary/congenital/Degenerative Chorioretinopathies	Pathologic myopia	58 (6.3%)
	Retinitis pigmentosa	20 (2.1)
	Chorioretinal coloboma	7 (0.76%)
	others	6 (0.65%)
	**Sub-total**	**91 (9.9%)**
**Optic Neuropathies**	Optic atrophy	10 (1%)
	Papilloedema	9 (0.98%)
	Indirect traumatic optic neuropathy	6 (0.65%)
	Papillitis	6(0.65%)
	**Sub-total**	**31(3.3%)**
**Grand Total**		**918 (100%)**

**Table 4:** The top ten vitreoretinal diseases diagnosed among patients presented to the University of Gondar Tertiary eye care and training center, vitreoretina clinic, NW Ethiopia. (n = 918).

s. No	Vitreoretinal disease	Frequency (%)
**1**	Diabetic Retinopathy	196(21.8%)
**2**	Age related macular degeneration	128(14%)
**3**	Rhegmatoginous retinal detachment	77(8.3%)
**4**	Pathologic myopia	58 (6.3%)
**5**	Hypertensive retinopathy	49 (5.3%)
**6**	Vitreous hemorrhage of any cause	49 (5.3%)
**7**	Retinal vein occlusions	45(5%)
**8**	Macular edema of any cause	42(4.5%)
**9**	Macular hole	31(3.3%)
**10**	Intermediate Uveitis	26(2.8%)

### Discussion

In this study, the mean age was 50.26 ±19.36 years and more than half of study patients, 441 (59.7%), were aged between 21 and 60 years. The mean age is comparable with a retrospective study done at Stanford University (USA) and a similar hospital based retrospective study done in South-eastern Nigeria in the University of Enugu, 52.2± 25.6 and 49.±16.8 year respectively but slightly higher than a prospective cross-sectional study done in Menillik-II tertiary eye care and training center in Ethiopia, 45.2±17.3 years and another Nigerian study 46.3 ± 21.4 years [7,9–11].

There was a male predominance in this study where nearly two-third (63.1%) of patients were male. This is in agreement with a study done in south eastern Nigeria (59.3%) and in Pakistan (62.8%) but against a study done in south western Nigeria, where 55.6% were females [11–13]. Factors contributing to male predominance in reports from developing countries are undoubtedly complex and it was suggested that men tend to seek medical attention more often than women in agricultural societies and this may certainly have contributed to the trend in our clinic [11,12].

In this study most of the patients, 434 (58.7%), came from urban area and this is similar to the mentioned hospital based cross-sectional study done Pakistan, 66.5% [7].

This study showed 220 (29.7%), 112 (16.2%) and 181(24.5%) patients had bilateral blindness, severe visual impairment and moderate visual impairment at presentation respectively. The rate of bilateral blindness in our study is higher than the previous similar study in Ethiopia 16.1%, a study done in South Eastern Nigeria 6.1% and a Nepalese study 12.26% [6,12,13]. This may be partly due to the fact that one third of our patients (33.5%) had cataract as an ocular comorbidity.

Ocular comorbidities were found in 633 (85.7%) of our patients with cataract 246(33.5%) blepharitis 173(24%) and glaucoma 52 (7%) being the most commonly diagnosed comorbidities. This finding is different from a hospital based retrospective study done in South-eastern Nigeria at the University of Enugu, where refractive error (19.8%) was the commonest but similar with a population based cross-sectional Tehran eye study where cataract (12.47%) was the most commonly diagnosed ocular comorbidity [9,13].

Systemic comorbidities were found in 325 (44%) of our patients with diabetes mellitus 196(26.5%) and hypertension 47(6.3%), being the most common. This is in agreement with a hospital based retrospective study done in South-eastern Nigeria and the Tehran eye study results where diabetes mellitus and hypertension were the two most common systemic comorbidities [9,13].

Our study showed Diabetic retinopathy, Age related Macular Degeneration (AMD) and Rhegmatoginous Retinal Detachment (RRD) were the top three retinal diseases diagnosed in 196 (22.44%), 128(14%) and 77 (8.4%) of our patients respectively. This result is similar with a hospital based study done in Pakistan which showed Diabetic related retinal conditions and Retinal detachment, were the commonest retinal pathologies seen in 39.8% and 20.6% of cases respectively. It is also in agreement with a hospital based study in south eastern Nigeria where diabetic retinopathy 24.9% was the commonest diagnosis. However, our finding was different from two other Nigerian Studies and a population based study in Nepal where Age Related Macular degeneration was the commonest diagnosis [7–9,11,14].

The pattern of retinal diseases seen in our study was comparable with the descriptive cross-sectional study done in Menillik-II tertiary eye care center in Ethiopia, where Retinal vascular diseases accounted for the largest group of patients (38.1%) of which diabetic retinopathy accounted for 75.1% but it differs in that Retinal detachment was the second largest group of diseases, accounting for 24.5% of the total. This is possibly due to the high referral burden rate of retinal detachment cases in Menillik-II Tertiary eye care center as it was a national referral center for surgical treatment of retinal detachment at the time of the study. The prevalence of AMD was 2.7% in this study and this is much lower than our study (14%) and this is possibly due to the fact that our study population was slightly older [7,10,11,15].

### Conclusion

Most of the vitreoretinal pathologies in our study were bilateral and males were affected more than females. The age group between 21–60 years accounted nearly two-third of study patients and this is the working age population suffering from vision loss from vitreoretinal diseases. Diabetic retinopathy, Age related macular degeneration and Rhegmatoginous Retinal detachment were the commonest retinal pathologies accounting for nearly half of the vitreoretinal diseases diagnosed and these conditions are treatable either medically or surgically. However, available facilities at the center are inadequate for the management of the most common vitreoretinal diseases. The vision losses at presentation of the study patients were much worse than other similar studies. Systemic and ocular comorbidities were also common findings among study patients. Strengthening the vitreoretina services of the University of Gondar tertiary eye care and training center with relevant equipment and other facilities is recommended.

## Supporting information

S1 Dataset (DOCX)
