# Supplementary material for: Pattern of vitreo-retinal diseases at University of Gondar tertiary eye care and training center, North-West Ethiopia
Source: PLoS One. 2022 Apr 21;17(4):e0267425. doi: 10.1371/journal.pone.0267425 (PMC9022795; doi:10.1371/journal.pone.0267425)
Supplement: S1 Dataset — (DOCX) [file pone.0267425.s001.docx]

Table I. Socio-demographic characteristics of patients with vitreo-retinal diseases presented to the University of Gondar Tertiary eye care and training center vitreoretina clinic, NW Ethiopia (n=739).

| variables | Categories | Frequency (%) |
| --- | --- | --- |
| Gender | Male | 466(63.1%) |
|  | Female | 273 (36.9%) |
| Age (years) | ≤20 | 66(9%) |
|  | 21-60 | 441(59.7%) |
|  | >60 | 337 (32.1%) |
| Occupation | Farmer | 269(37%) |
|  | Gov’t employee | 174(23.4%) |
|  | student | 64(8.7%) |
|  | Private employee | 55(7.4%) |
|  | Merchant | 40(5.4%) |
|  | Other | 137(19%) |
| Marital status | Married | 526(71.2%) |
|  | Single | 130(17.6%) |
|  | Divorced | 83(11.2%) |
| Religion | Christian | 627 (84.8%) |
|  | Muslim | 112(15.2%) |
| Annual Income (USD) | <200 | 455(61.6%) |
|  | 200-600 | 167(22.6%) |
|  | >600 | 117(15.8%) |
| Residence | Rural | 305(41.3%) |
|  | Urban | 434(58.7%) |

Table II. Clinical characteristics of patients with vitreo-retinal diseases presented to the University of Gondar Tertiary eye care and training center vitreoretina clinic, NW Ethiopia. (n=739)

| Variables | Categories | Frequency (%) |
| --- | --- | --- |
| Presenting complaint | Reduction of vision | 519(70.2%) |
|  | Flashlights and floaters | 47 (6.35%) |
|  | Came for routine check up | 118(16%) |
|  | Others | 55(7.4%) |
| Duration of complaint | >1 year | *290(39.2%)* |
|  | 6 month -1 year | *99 (13.4%)* |
|  | 1month to <6month | *124(16.8%)* |
|  | < 1 month | *108(14.6%)* |
| Visual Acuity at presentation in the better eye | ≥6/18 | 220(29.8%) |
|  | 6/24-6/60 (MVI)* | 181(24.5%) |
|  | <6/60-3/60 (SVI)* | 112(16.2%) |
|  | <3/60 (Blind) | 220(29.7%) |
| Laterality | Unilateral disease | 235(21.8%) |
|  | Bilateral disease | 504(68.2%) |
| Systemic co-morbodities | Diabetes Mellitus | 206(27.8%) |
|  | Hypertension | 47(6.3%) |
|  | Hyperlipidemia | 21 (2.8%) |
|  | Neurologic diseases | 19(2.6%) |
|  | Cardiac diseases | 14(1.9%) |
|  | Pulmonary diseases | 13 (1.8%) |
|  | HIV/AIDS | 12(1.6%) |
|  | Others | 23(3.1%), |
| Ocular comorbidities | Cataract | 246(33.5%) |
|  | Blepharitis | 173 (24%) |
|  | Glaucoma | 52 (7%) |
|  | Refractive error | 35(4.7%) |
|  | others | 97 (13%) |

*MVI-Moderate Visual impairment

*SVI-Severe visual impairment

Table III. Category and vitreoretinal diagnoses made among patients presented to the University of Gondar Tertiary eye care and training center, vitreo-retina clinic, Ethiopia. (N=918)

| Disease category | Diagnosis | Frequency (%) |
| --- | --- | --- |
| Retinal vascular diseases | Diabetic retinopathy | 196(21.3%) |
|  | Hypertensive retinopathy | 49(5.3%) |
|  | Branch or central Retinal vein occlusion | 45(5%) |
|  | Coats disease | 3(0.3%) |
|  | Idiopathic parafoveal telangiectesia | 1(0.1%) |
|  | **Sub-total** | **304 (33.1%)** |
| Macular diseases | Age related macular degeneration | 128(14%) |
|  | Macular edema of any cause | 42(4.5%) |
|  | Macular Hole | 31(3.3%) |
|  | Non-specific macular scar | 14 (1.5%) |
|  | Choroidal neovascularization (Non-AMD) | 3 (0.3%) |
|  | Macular dystrophies | 3(0.3%) |
|  | Epiretinal membrane | 3(0.3%) |
|  | **Sub-total** | **224 (24.4%)** |
| Retinal detachments | Rhegmatoginous retinal detachment | 77(8.3%) |
|  | Exudative Retinal detachment | 4(0.4%) |
|  | Tractional Retinal detachment | 11(0.1%) |
|  | **Sub-total** | **92 (10%)** |
| Vitreous pathologies | Vitreous hemorrhages of any cause | 49 (5.3%) |
|  | Posterior vitreous detachment | 18(1.9%) |
|  | Asteroid hyalosis | 16(1.7%) |
|  | others | 4(0.4%) |
|  | **subtotal** | **87 (9.4%)** |
| Inflammatory/Infectious/Traumatic Chorioretinopathies | Intermidiate uveitis | 26(2.8%) |
|  | idiopathic inflammatory retinochoroiditis | 21(2.3%) |
|  | Traumatic chorioretinopathies | 20 (2.1%) |
|  | Infectious retinochoroiditis | 14(1.5%) |
|  | Idiopathic Retinal vasculitis | 8(0.9%) |
|  | **Sub-total** | **89 (9.6%)** |
| Hereditary/congenital/Degenerative  Chorioretinopathies | Pathologic myopia | 58 (6.3%) |
|  | Retinitis pigmentosa | 20 (2.1) |
|  | Chorioretinal coloboma | 7 (0.76%) |
|  | others | 6 (0.65%) |
|  | **Sub-total** | **91 (9.9%)** |
| Optic Neuropathies | Optic atrophy | 10 (1%) |
|  | Papilloedema | 9 (0.98%) |
|  | Indirect traumatic optic neuropathy | 6 (0.65%) |
|  | Papillitis | 6(0.65%) |
|  | **Sub-total** | **31(3.3%)** |
| Grand Total | | **918 (100%)** |

Table-IV. The top ten vitreoretinal diseases diagnosed among patients presented to the University of Gondar Tertiary eye care and training center, vitreoretina clinic, NW Ethiopia. (n=918)

| **s. No** | **Vitreoretinal disease** | **Frequency (%)** |
| --- | --- | --- |
| **1** | Diabetic Retinopathy | 196(21.8%) |
| **2** | Age related macular degeneration | 128(14%) |
| **3** | Rhegmatoginous retinal detachment | 77(8.3%) |
| **4** | Pathologic myopia | 58 (6.3%) |
| **5** | Hypertensive retinopathy | 49 (5.3%) |
| **6** | Vitreous hemorrhage of any cause | 49 (5.3%) |
| **7** | Retinal vein occlusions | 45(5%) |
| **8** | Macular edema of any cause | 42(4.5%) |
| **9** | Macular hole | 31(3.3%) |
| **10** | Intermediate Uveitis | 26(2.8%) |
